# Sustained intra-articular reactive oxygen species scavenging and alleviation of osteoarthritis by biocompatible amino-modified tantalum nanoparticles

**DOI:** 10.3389/fbioe.2023.1118850

**Published:** 2023-01-13

**Authors:** Yunsheng Jiang, Tao Li, Junjun Yang, Xin Wang, Xiongbo Song, Guangxing Chen, Gang Dai, Rong Li, Chunyan Yao, Jiajia Chen, Cheng Chen, Xiaoyuan Gong, Liu Yang

**Affiliations:** ^1^ Center for Joint Surgery, Southwest Hospital, Third Military Medical University (Army Medical University), Chongqing, China; ^2^ State Key Laboratory of Trauma, Burns and Combined Injury, Chongqing Engineering Research Center for Nanomedicine, Institute of Combined Injury, College of Preventive Medicine, Third Military Medical University (Army Medical University), Chongqing, China; ^3^ Key Laboratory of Biorheological Science and Technology, Ministry of Education, College of Bioengineering, Chongqing University, Chongqing, China; ^4^ Blood Transfusion Department, Southwest Hospital, Third Military Medical University (Army Medical University), Chongqing, China; ^5^ Biomedical Analysis Center, Third Military Medical University (Army Medical University), Chongqing, China; ^6^ College of Medical Informatics, Chongqing Medical University, Chongqing, China

**Keywords:** tantalum nanoparticles, catalase activity, ROS scavenging agent, oxidative stress, osteoarthritis

## Abstract

Recent studies highlight the vital role of oxidative stress and reactive oxygen species (ROS) during progression of osteoarthritis (OA). Attenuating oxidative stress and reducing reactive oxygen species generation in joints represent reasonable strategies for the treatment of osteoarthritis. To address the potential question for clinical translation, and improve the biocompatibility and long-term performance of current antioxidants, the present study provided high biocompatible small positively charged tantalum nanoparticles (Ta-NH_2_ NPs) with sustained intra-articular catalase activity and first applied to osteoarthritis intervention. Our *in vitro* results showed that Ta-NH_2_ NPs were stable with good biocompatibility, and protected viability and hyaline-like phenotype in H_2_O_2_-challenged chondrocytes. In addition, the *in vivo* biodistribution data demonstrated a sustained retention of Ta-NH_2_ NPs in the joint cavity, particularly in articular cartilage without organ toxicity and abnormality in hemogram or blood biochemistry indexes. Finally, compared with catalase (CAT), Ta-NH_2_ NPs exhibited long-term therapeutic effect in monosodium iodoacetate (MIA) induced osteoarthritis model. This study preliminarily explored the potential of simply modified metal nanoparticles as effective reactive oxygen species scavenging agent for osteoarthritis intervention, and offered a novel strategy to achieve sustained reactive oxygen species suppression using biocompatible Ta-based nano-medicine in oxidative stress related diseases.

## Introduction

Osteoarthritis (OA) is the most common form of joint diseases with manifestations of chronic joint inflammation (Tamaddon et al., 2020), and may result in pain, joint malformation and limited mobility in patients (Quicke et al., 2022). OA is the leading cause of long-term disability in adults, and the prevalence of OA is estimated to reach 40%. OA is characterized by morphological, biochemical, molecular and biomechanical changes of both cells and extracellular matrix (ECM) in articular cartilage, synovium, and subchondral bone (Tamaddon et al., 2020). Although the mechanisms of OA initiation and progression are yet to be understood, recent studies have highlighted the vital role of oxidative stress and reactive oxygen species (ROS) in mitochondrial dysfunction (Yao et al., 2021), chondrocyte senescence, matrix synthesis (Rahmati et al., 2017), synovial inflammation (Sanchez-Lopez et al., 2022), and subchondral bone dysfunction during progression of OA. Oxidative stress has been defined as a disturbance in the balance between the production of ROS and antioxidant defences, which results in macromolecular damage and disruption of redox signalling (Yang et al., 2020). Oxidative stress amplifies inflammatory responses and exacerbates cartilage breakdown (Yang et al., 2022). Attenuating the oxidative stress level by antioxidants drugs or natural antioxidants could effectively decrease the progression of OA in animal models (Gui et al., 2022). Therefore, attenuating oxidative stress and reducing ROS generation in joints represent promising strategies for the treatment of OA.

Currently, antioxidant supplementations (Gui et al., 2022), mediators of various ROS pathways (Yang et al., 2020), and free radical scavengers have been successively utilized to target oxidative stress in the pathogenesis of OA (Guillén et al., 2021). Among them, the commonly used antioxidants are simply divided into macro and small molecules. However, systemic application of these antioxidants might related to poor safety, pharmacodynamics, and bioavailability (Brown et al., 2019). ROS scavenging therapy for OA is thus administered by intra-articular injection. However, in addition to instability of these antioxidant caused by fluctuation in osteoarthritic physicochemical microenvironment, small molecules are rapidly cleared from the joint within hours *via* the synovial vasculature, and large molecules are cleared through the synovial lymphatic vessels within days (Gerwin et al., 2006). Therefore, high-dosage injections are often required to achieve the desired working concentration (Gammer and Brobäck, 1984). To overcome these shortcomings and prolong the action of antioxidant for further clinical application, inorganic metal nano-enzyme such as ultrafine copper oxide (Liu T. et al., 2020) and Fe-curcumin nanozyme (Yuan et al., 2022) were developed and have displayed good bioavailability and excellent therapeutic efficiency at very low concentration (≈nM/mL). However, the potential disadvantages such as ionization in body fluid (Matusiewicz, 2014) and long-term metal cumulation of toxic side effects (Ring et al., 2016; Badhe et al., 2021) still impede its clinical application.

Tantalum (Ta) is an inert metal with strong corrosion resistance and excellent biocompatibility (Levine et al., 2006; Dou et al., 2019). Ta-based implantations are widely used in joint replacement and bone defect repair. Unlike cobalt and titanium, Ta-based implants have been reported to maintain an excellent long-term biocompatibility (Levine et al., 2006). In addition to the application in tissue engineering, tantalum nanoparticles (Ta-NPs) have also gained reasonable attention in early diagnosis and photothermal therapy of malignant tumour due to its high biocompatibility. For instance, recent studies have demonstrated that tantalum oxide NPs are an excellent contrast agent for computed tomography (CT) (Oh et al., 2011). In addition, 2D Ta carbide (Dai et al., 2017) and Ta carbide nanosheets (Liu et al., 2018) have been explored as light-induced therapeutic agents for tumour diagnosis and therapy. Interestingly, studies also suggest that Ta-coated surfaces could effectively alleviate oxidative stress induced by a high-glucose environment in a diabetes model (Wang et al., 2016), what’s interesting is that the some research reported the similar properties to catalase of Ta-NPs (Miao et al., 2019). The aforementioned properties endow Ta-NPs with the ability to effectively alleviate oxidative stress by eliminating H_2_O_2_-induced ROS, and make it possible for its application in the treatment of OA.

**FIGURE 1:**
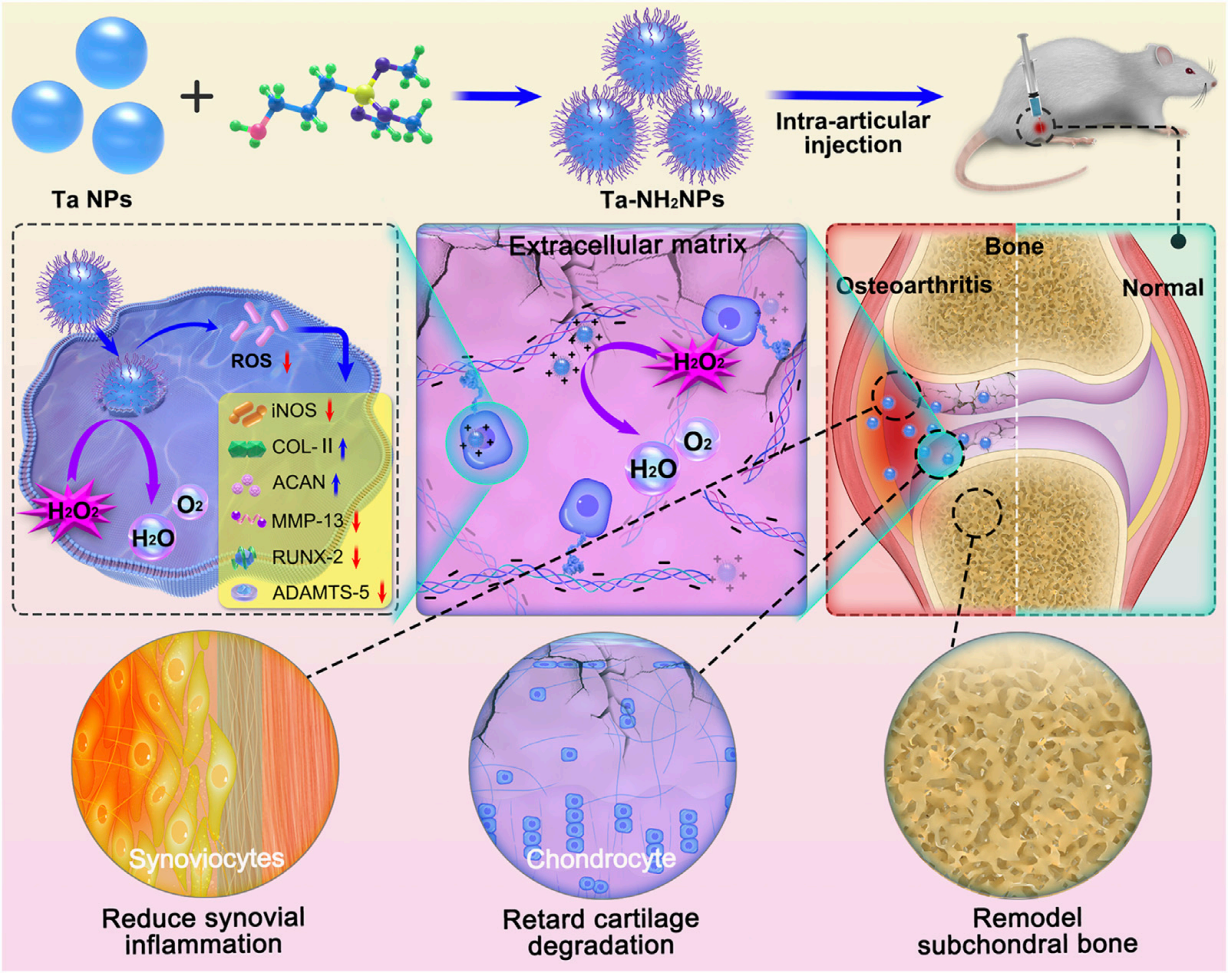
Schematic diagram of the synthesis of Ta-NH_2_ NPs and the treatment of OA with single intra-articular injection. Positively charged NPs obtained by adding amino groups to the surface of Ta was injected into the osteoarthritic joint cavity. Ta-NH_2_ NPs exhibit long-term oxidative stress attenuating effect in articular cartilage, and alleviates OA progression.

Based on the mentioned advantages of Ta, the present study designed Ta-NPs with amino-modification for OA-related oxidative stress modulation. To highlight the potential for clinical translation, the surface of Ta-NPs was modified by amino group (Ta-NH_2_ NPs) with silane-coupling approach. The positive charged Ta-NPs were designed to retain in negatively charged cartilaginous ECM (Brown et al., 2019), enabling the continuous and steady regulation of the oxidative stress level in osteoarthritic joint. We hypothesize that the Ta-NH_2_ NPs alleviate OA progression in monosodium iodoacetate (MIA)-induced OA rat model (Figure 1). The *in vitro* antioxidative effect of Ta-NPs was evaluated by hydrogen peroxide decomposition analysis and ROS staining. The regulation of Ta-NH_2_ NPs on chondrocyte phenotype was verified by western blotting and immunostaining experiments. In addition, near-infrared fluorescence *in vivo* imaging, complete blood panel analysis and serum biochemistry test, micro-CT and histological analysis were employed to verify the efficiency, safety and therapeutic effect of Ta-NPs after intra-articular injection.

## Materials and methods

### Materials

Ta NPs, collagenase II and hydrogen peroxide solution were purchased from Sigma (United States). Catalase, Alcian blue staining solution, Crystal violet staining solution, Hydrogen peroxide detection kit, Reactive oxygen species assay kit, Phosphate-buffered saline (PBS), Penicillin-streptomycin solution (P/S), 4% paraformaldehyde fix solution, immunostaining permeabilization buffer with Triton X-100, QuickBlock primary/secondary antibody dilution buffer for immunofluorescence/Western blot, immunol staining/Western blot wash buffer, QuickBlock blocking buffer, soaking and activation buffer for polyvinylidene fluoride (PVDF) membrane, SDS-PAGE gel preparation kit, BCA protein assay kit, and DAPI staining solution were obtained from Beyotime (China). Absolute ethyl alcohol was gained from MACKLIN (China). DMEM/High glucose medium, fetal bovine serum (FBS), trypsin, and dimethyl sulfoxide (DMSO) were purchased from Gibco (United States). (3-Aminopropyl) trimethoxysilane was purchased from Aladdin (China). Cell counting kit-8 was purchased from Dojindo Laboratories (Japan). Calcein-AM/PI double stain kit was purchased from Bestbio (China). The primary antibody against collagen I (COL-I), collagen II (COL-II), aggrecan (ACAN), SOX-9, iNOS, ADAMTS-5, MMP-13, β-action were purchased from Affinity (China). Goat Anti-Rabbit IgG H&L (Alexa Fluor^®^488) was purchased from Abcam (United States). Ethylene Diamine Tetraacetic Acid (EDTA) was purchased from Biosharp (China). CY5.5 staining were purchased from Ruixi (China). All chemicals were of reagent grade.

### Surface amino modification of Ta NPs

The surface of Ta NPs was modified with amino group using the silane-coupling method. Briefly, Ta NPs (1 g) were dispersed in ethanol (10 mL) under tip ultrasound for 20 min, followed by centrifugation (800 g, 5 min) to remove large sized NPs. The concentration of collected solution was determined with oven drying method and diluted to 1 mg/mL with ethanol. (3-Aminopropyl) trimethoxysilane (100 μL) was added into Ta NPs (20 mL) and stirred for another 3 h at 70°C. Finally, Ta-NH_2_ NPs were obtained by centrifugation (12,000 g, 5 min) and washed with deionized water to remove the free (3-Aminopropyl) trimethoxysilane for three times.

### Characterization of Ta and Ta-NH_2_ NPs

The morphology of Ta NPs and Ta-NH_2_ NPs was observed using Hitachi S-3400NIISEM (Hitachi, Japan) at 2 kV accelerating voltage. Transmission electron microscope (TEM) and element mapping images were conducted by JEM 2100F (JEOL, Japan). The hydrodynamic sizes, particle dispersity index (PDI), and zeta-potential of Ta-NH_2_ NPs were analyzed with the Malvern Zetasizer Nano ZS instrument (Malvern, United Kingdom). Fourier transforms infrared (FT-IR) spectra of Ta-NH_2_ NPs were measured using Nicolet IS 10 spectrometer (Nicolet, United States). X-ray diffraction (XRD) of Ta-NH_2_ NPs was detected by an X-ray diffractometer D8 ADVANCE (Bucker, Germany) with the 2-Thera range from 10° to 90°. X-ray photoelectron spectroscopy (XPS) of Ta-NH_2_ NPs was determined by an X-ray photoelectron spectrometer 250Xi (Thermo Fisher, United States).

### H_2_O_2_ scavenging activity assay

H_2_O_2_ scavenging activity of Ta-NH_2_ NPs was tested by the Hydrogen Peroxide Detection Kit. Ta-NH_2_ NPs with different concentrations (0, 10, 20, 50, and 100 μg/mL) were incubated with 1 mM H_2_O_2_ at 37°C for 1 h, respectively. After reaction, the concentration of remained H_2_O_2_ was determined according to the manufacturer’s instructions, and the scavenging capacity was calculated. For assessment of Ta-NH_2_ NPs with stable H_2_O_2_ scavenging activity, Ta-NH_2_ NPs (100 μg/mL) were incubated with 1 mM H_2_O_2_ under different temperature (25°C, 37°C, 50°C, and 60°C) and pH (2, 4, 6, 7, 8, and 10), respectively.

### Isolation of chondrocyte and cell culture

All animal experiments were performed in accordance with the guidelines approved by the Laboratory Animal Welfare and Ethics Committee of Army Medical University (Chongqing, China). The methods of chondrocytes isolation were in accordance with the previously published article. Male Sprague-Dawley (SD) rats (200–300 g) were suffocated by CO_2_, and knee joint was dissected. The cartilage was broken up with ophthalmic scissors and digested by type II collagenase for 12 h. After filtering out cartilage residue, primary chondrocytes were obtained. The obtained chondrocytes were cultured with Dulbecco’s modified Eagle’s medium (DMEM) supplemented with 10% fetal bovine serum (FBS), 100 μg/mL streptomycin, and 100 U/mL penicillin at 37°C in 5% CO_2_ incubator. The experimental cells were selected as P2 generation rat chondrocytes.

### *In vitro* cytotoxicity evaluation of Ta-NH_2_ NPs

The cytotoxicity of Ta-NH_2_ NPs was determined by the CCK-8 assay *in vitro*. Briefly, chondrocytes (1 × 10^4^ per well) were seeded into 96-well culture plates and incubated at 37°C in an 5% CO_2_ incubator for 24 h. Different concentrations of Ta-NH_2_ NPs (0, 5, 10, 20, 50, 100, 200, and 500 μg/mL) were added to each well and incubated for another 24 h or 48 h. The cell viability was then quantified by measuring the absorbance value at 450 nm with microplate reader.

### Detection of intracellular ROS

Chondrocytes (1 × 10^5^ per dish) were seeded into confocal petri dish and pre-treated with Ta-NH_2_ NPs (100 μg/mL) for 2 h. 400 μM H_2_O_2_ was then added into petri dish and cells were incubated for another 24 h. After washing with PBS, cells were stained with DCFH-DA probes following the manufacturer’s instructions and subjected to flow-cytometry detection.

### Immunofluorescence staining

The above-described cells were washed with PBS, and fixed with 4% paraformaldehyde, followed by blockage with Quick Blocking buffer. Next, the cells were incubated with primary antibody (COL-I, COL-X, COL-II, SOX-9, ACAN, MMP-13, ADAMTS-5, and RUNX-2) and then with secondary antibody. Nuclei were stained with DAPI. The fluorescence images were observed with laser scanning confocal microscope, and the relative fluorescence intensity was analyzed with ImageJ (version 1.52p). The immunofluorescence staining work concentration of COL-I, COL-II, ACAN, SOX-9, iNOS, ADAMTS-5 and MMP-13 was 1:100. The immunofluorescence staining work concentration of Goat Anti-Rabbit IgG H&L (Alexa Fluor^®^488) was 1:200.

### Western blot analysis

The total proteins were isolated from above-described cells using RIPA lysis, and were quantified using BCA protein kit according to the manufacturer’s instructions. The protein was separated by SDS PAGE and subsequently transferred to PVDF membrane. Next, the cell lysis was incubated with primary antibody (COL-I, COL-X, COL-II, SOX-9, ACAN, MMP-13, ADAMTS-5, and RUNX-2), followed by secondary antibody incubation. Finally, the membranes were visualized using ultrasensitive ECL and the intensity of blots was quantified with Image Lab software (version 3.0). The WB work concentration of COL-I, COL-II, ACAN, SOX-9, iNOS, ADAMTS-5 and MMP-13 was 1:1000, and β-action was 1:10,000. Goat Anti-Rabbit IgG H&L (HRP) WB work concentration was 1:5000.

### Fluorescence *in vivo* imaging

To determine the retention time of Ta-NH_2_ NPs in the articular joint, female SD rats’ (250–300 g) knees were injected with 100 μL of Ta-NH_2_ NPs-CY5.5 (100 μg/mL). The fluorescence images were captured at various time points (1, 3, 7, 14, and 28 d) using *In Vivo* Imaging System Pearl Trilogy (LI-COR, United States). The knee joint, femur condyles, and major organs (heart, liver, spleen, lung, kidney, and brain) were harvested for *ex vivo* NIR imaging. The relative fluorescence intensities were analyzed using Image Studio (version 5.2).

### *In vivo* toxicity evaluation of Ta-NH_2_ NPs

Blood samples were harvested at day 1 and 28 after intra-articular injection of 100 μL of Ta-NH_2_ NPs. The serum biochemistry tests included two important indicators of hepatic function: aspartate aminotransferase (AST) and alanine aminotransferase (ALT), and two indicators of kidney function: blood urea nitrogen (BUN) and creatinine (CRE). Complete blood count was done to further evaluate the *in vivo* toxicity of Ta-NH_2_ NPs. Furthermore, the harvested major organs (liver, heart, spleen, lung, kidney, and brain) were subjected to hematoxylin and eosin (H&E) staining and histological analysis.

### MIA-induced OA model and Ta-NH_2_ NPs intraarticular injection

The rat OA model was induced by intra-articular injection 20 μL of MIA (2 mg/mL). The next experiment will be conducted 2 weeks after injection. Rats were randomly divided into 4 groups (*n* = 8): Control group (Intra-articular injection of PBS), MIA group (Intra-articular injection of MIA), MIA + Ta-NH_2_ NPs group (Intra-articular injection of MIA, followed by Ta-NH_2_ NPs treatment), and MIA + CAT group (Intra-articular injection of MIA, followed by CAT treatment). Rats were sacrificed after treatment with Ta-NH_2_ NPs or CAT for 4 or 8 weeks. The knee joints were collected and fixed in 4% paraformaldehyde.

### Micro CT imaging

The knee joints were scanned with micro-CT (viva CT-40, ScancoMedical AG, Switzerland). Image acquisition was performed with the condition of 45 kV and 177 μA in high-resolution scans (10.5 μm voxel resolution). Two-dimensional images were used to generate three-dimensional reconstructions. The epiphysis of the tibial subchondral bone was manually chosen as the region of interest during three-dimensional analysis (micro-CT Evolution Program V6.5 software). The three-dimensional parameters of trabecular bone, including bone volume fraction (BV/TV, %), trabecular thickness (Tb.Th, mm). Interest area (10 mm, 10 mm, 5 mm) was selected with software, and this area was limited to the upper surface of the specimens.

### Histological analysis

The knee joints were decalcified in 10 wt% ethylene diamine tetraacetic acid (EDTA) solution for 4 weeks at 25°C. To observe the degeneration of knee articular cartilage, synovial inflammation and corresponding inducible nitric oxide synthases (iNOS) changes, H&E, Safranin-o-fast green staining, and immune-histochemical staining against iNOS were performed respectively. Three independent experts were asked to perform double-blind scoring according to OA Research Society International (OARSI) scoring system.

### Statistical analysis

Graphical results were displayed as mean ± s.d by using GraphPad prism software (version 7.0). All data were assessed for normality using the Kolmogorov-Smirnov test and for homoscedasticity using the F-test. The statistical significance difference between two groups was compared by independent-sample *t*-test for parametric data, and by Mann-Whitney test for non-parametric data. The Welch’s correction was applied for variables with unequal variance. The statistical significance differences bet ween vehicle and other treatment groups were determined by One-way ANOVA test and Fisher’s LSD post-test for parametric data, and by Kruskal Wallis test and Dunn’s multiple comparisons post-test for non-parametric data. In all cases, statistical significance was defined with *p* < 0.05.

## Results and discussion

### Ta-NH_2_ NPs synthesis and characterization

**FIGURE 2:**
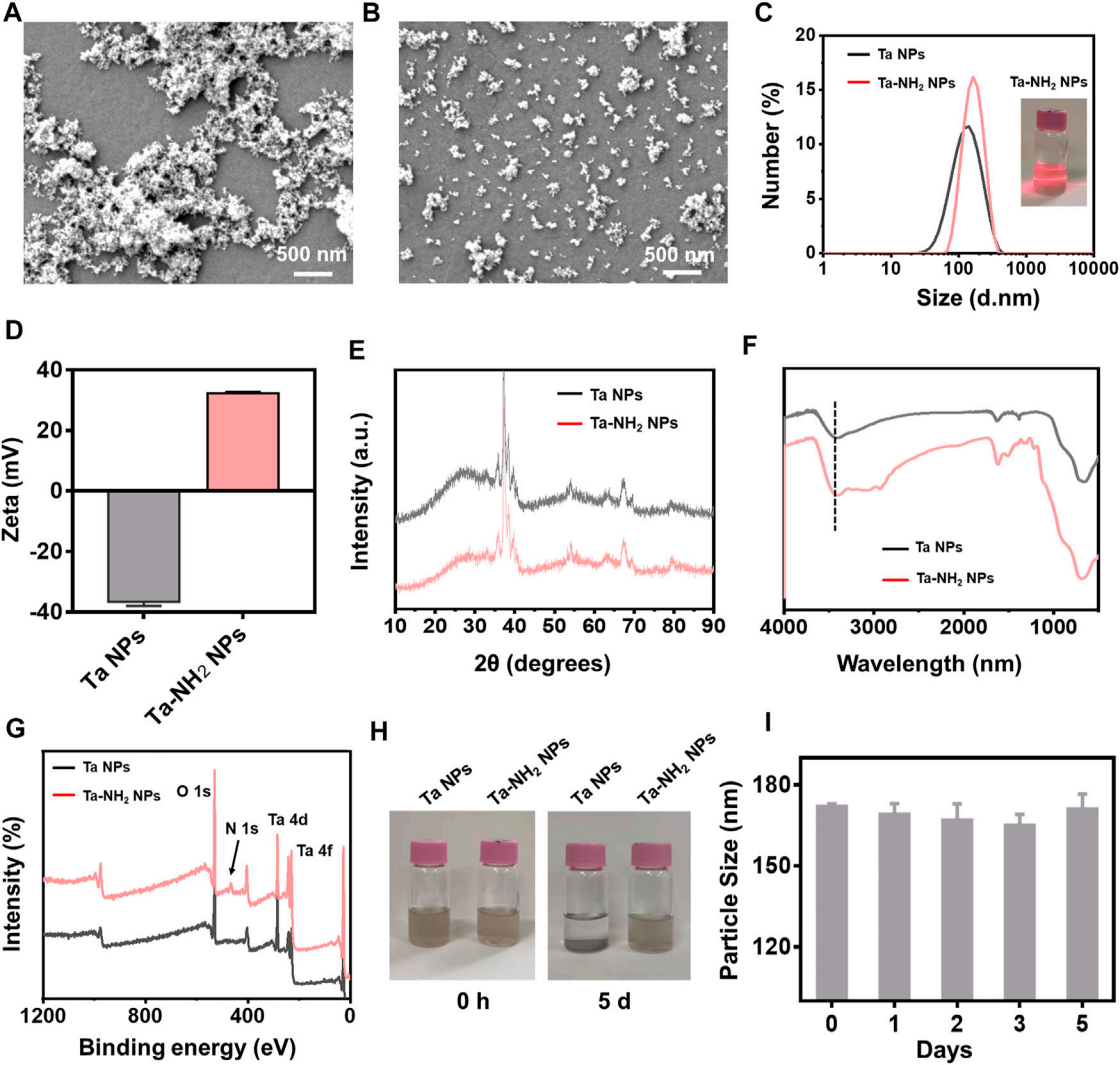
Morphological and compositional characterizations of Ta-NH_2_ NPs. **(A)** SEM image of Ta NPs and **(B)** SEM image of Ta-NH_2_ NPs. **(C)** Hydrodynamic diameters and **(D)** Zeta potential of Ta NPs and Ta-NH_2_ NPs measured by DLS. **(E)** XRD patterns and **(F)** FT-IR spectra of Ta NPs and Ta-NH_2_ NPs. **(G)** XPS spectra of Ta-NH_2_ NPs, **(H)** Representative digital photographs of Ta NPs and Ta-NH_2_ NPs at 0 d and 5 d. **(I)** Hydrodynamic diameters of Ta-NH_2_ NPs measured by DLS at 1–5 d.

Pristine Ta NPs with high density have poor colloidal stability in common aqueous (Miao et al., 2019). To improve the colloidal stability of Ta NPs and increase their retention in negatively charged articular cartilage ECM (Freedman et al., 2014), the surface of Ta NPs was modified with amino group. Ta-NH_2_ NPs were synthesized by a simple and efficient silane-coupling approach, which is widely used for modification of the surface of inorganic NPs (Wu et al., 2020). In detail, the commercial raw Ta NPs were dispersed in ethanol and then centrifugated to separate large-sized NPs. Amino groups were coated on the surface of Ta NPs by refluxing reaction with (3-Aminopropyl) trimethoxysilane in ethanol. From scanning electron microscopy (SEM) (Figures 2A, B) images, Ta-NH_2_ NPs exhibited uniform monodispersed compared with Ta NPs. Meanwhile, in consistent with previous report (Miao et al., 2019), as shown in the TEM image (Supplementary Figure S1A), the Ta NPs and Ta-NH_2_ NPs were observed with irregular elliptical morphology. HR-TEM elemental mapping images (Supplementary Figure S1B) indicated that Ta, O, and N elements were uniformly distributed in Ta-NH_2_ NPs. Whereas only elemental Ta and O emerged in Ta NPs, verifying the successful modification of amino group on Ta NPs surface. As shown in Figure 2C, the hydrodynamic sizes of Ta NPs and Ta-NH_2_ NPs determined by dynamic light scattering (DLS) were 116.73 ± 0.31 and 168.77 ± 8.04 nm with a narrow PDI (0.18 ± 0.01 vs. 0.16 ± 0.01), respectively. Meanwhile, the zeta potential changed from negative (Ta NPs, −36.63 ± 1.31 mV) to positive charge (Ta-NH_2_ NPs, 32.27 ± .51 mV) (Figure 2D), which could be explained by the modification of Ta NPs with amino group ligands spreading out in solutions. As shown in the XRD patterns (Figure 2E), Ta-NH_2_ NPs maintained all the characteristic peaks of the Ta NPs at high angles of 30°-80°, indicated that the modification of amino group on Ta NPs surface did not decrease the crystalline purity of Ta NPs. In addition, from FT-IR spectra (Figure 2F), the Ta-NH_2_ NPs appeared new characteristic bands around 3,300 cm^−1^ of -NH_2_, confirming that amino group had been successfully bonded on the surface of Ta NPs. In addition, the chemical valence of Ta_2_O_5_ and metallic Ta were detected at 28 eV (Ta_4_f_7/2_) and 26 eV (Ta_4_f_5/2_) from the XPS spectra (Figure 2G), which could be attributed to partial oxidation on the surface of Ta NPs and turned into a more stable form (Tsuchiya et al., 2011). The oxygenic groups on metal surface were beneficial to the reaction of silane with surface oxygenic groups of Ta NPs. Meanwhile, the chemical valence of N elements also could be observed at 470 eV (N1s) from the XPS spectra, further confirming the successful modification of NH_2_ on Ta NPs surface. As shown in Figure 2H, Ta-NH_2_ NPs exhibited stable dispersion in H_2_O for 5 days. Furthermore, no obvious hydrodynamic particle size change was observed in Ta-NH_2_ NPs after incubation in H_2_O for 5 days, indicating a good colloidal stability (Figure 2I). Taken together, our data demonstrated that amino groups had been successfully decorated on the surface of Ta NPs with improved colloidal stability.

### H_2_O_2_ scavenging activity of Ta-NH_2_ NPs

**FIGURE 3:**
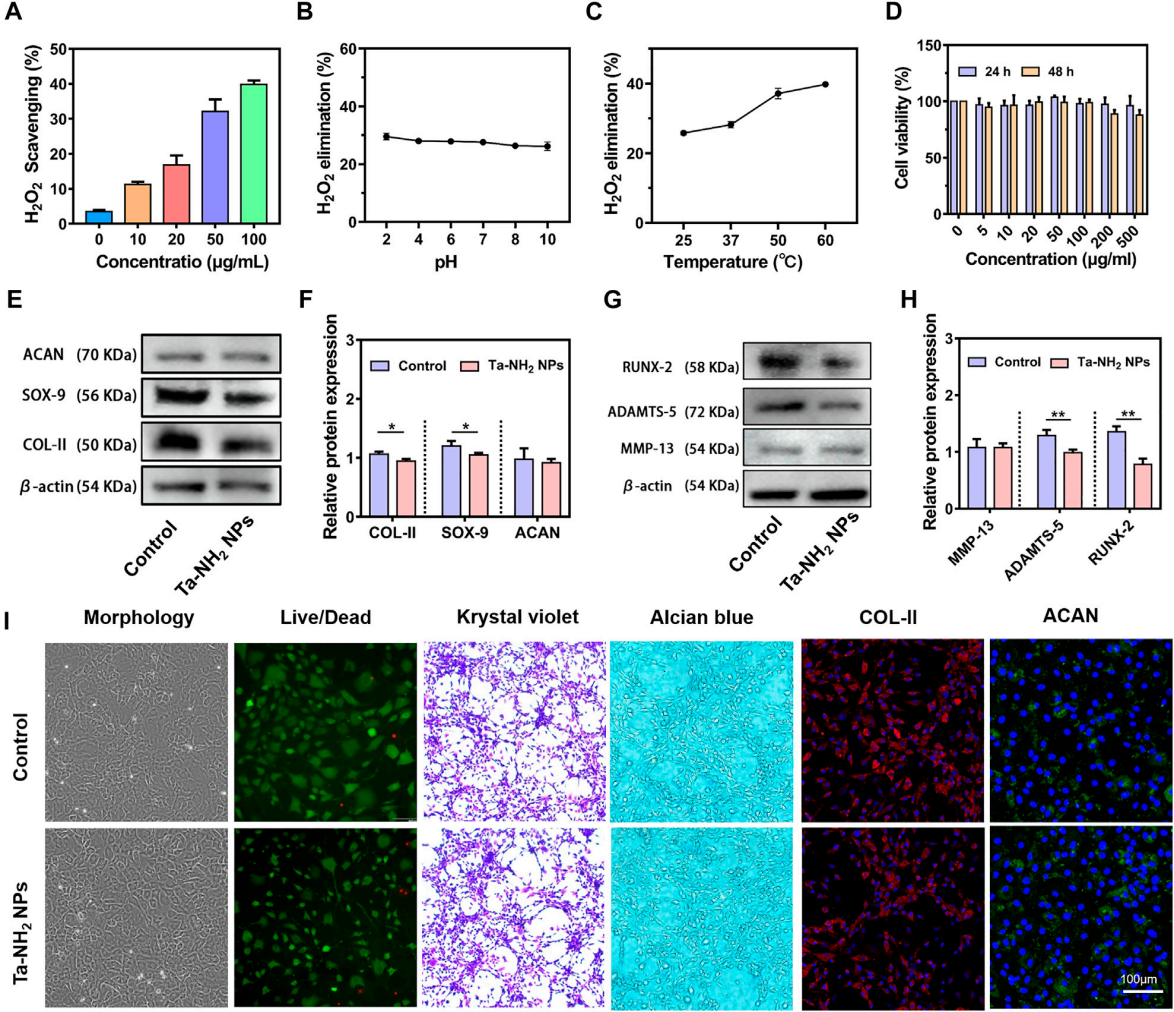
H_2_O_2_ scavenging activity and *in vitro* biocompatibility of Ta-NH_2_ NPs. **(A)** Concentration dependent H_2_O_2_ scavenging activity of Ta-NH_2_ NPs during 1 h incubation. **(B)** pH and **(C)** temperature stabilities of H_2_O_2_ scavenging activity in Ta-NH_2_ NPs (100 μg/mL). **(D)** Chondrocyte viability under different Ta-NH_2_ NPs concentration treatments. **(E,F)** Western blot analysis of hyaline (COL-II, SOX-9, and ACAN), **(G,H)** hypertrophic and catabolic (MMP-13, RUNX-2, and ADAMTS-5) phenotypes of chondroyte after 24 h of Ta-NH_2_ NPs treatment. **(I)** Morphology, Live/Dead Krystal violet, Alcian blue, and COL-II/ACAN immunofluorescence staining of chondrocytes treated with Ta-NH_2_ NPs. Data were represented as means ± s.d, from three independent replicates. **p < 0.01.

H_2_O_2_, the representative ROS was selected to investigate the ROS scavenging activity of Ta-NH_2_ NPs *in vitro*. As shown in Figure 3A, 1 mM H_2_O_2_ was reacted with different concentration of Ta-NH_2_ NPs at 37°C for 1 h. Ta-NH_2_ NPs exhibited ROS scavenging activity in a concentration-dependent manner. Approximately 35% of the H_2_O_2_ was decomposed by 50 μg/mL Ta-NH_2_ NPs, and almost 40% H_2_O_2_ could be scavenged in the concentration 100 μg/mL with excellent pH and temperature stabilities (Figures 3B, C). When Ta-NH_2_ NPs were co-cultured with H_2_O_2_, a large number of bubbles were generated in the tube during H_2_O_2_ decomposition due to the surface effect of Ta-NPs, or similar to the internal electronic transition of cerium oxide, while the specific mechanism is worthy of further exploration and study (Supplementary Figure S2A). Compared with the working concentrations of reported metal NPs, recently reported ultrasmall copper oxide (Liu T. et al., 2020) and manganese dioxide NPs (Kumar et al., 2019) might manifest better catalytic efficiency than Ta-NH_2_ NPs. However, these reported metal nanomaterials might be ionized by body fluid to generate free metal ions, which further led to element imbalance or even metal poisoning (Ring et al., 2016; Badhe et al., 2021). In addition, compared with classical metal such as gold NPs (Li et al., 2017), Ta-NH_2_ NPs showed stronger H_2_O_2_ decomposition. In the present study, compared with traditional antioxidants, the stability analysis indicated that the H_2_O_2_ scavenging of Ta-NH_2_ NPs was not influenced by pH or temperature. It is well known that the activities of ROS scavenging in traditional biological enzymes were affected by the microenvironment of joint cavity, and this drawback might affect the therapeutic outcome due to the fluctuation in physical and chemical properties of osteoarthritic joints.

### The biocompatibility of Ta-NH_2_ NPs *in vitro*

The biocompatibility of Ta-based materials has been widely validated in orthopedic implants. In the present study, our *in vitro* biocompatibility analysis suggested that Ta-NH_2_ NPs also shared the same characteristics in bio-safety. The CCK-8 assay results showed no significant cytotoxicity at test concentration after 24 and 48 h co-culture (Figure 3D). But slightly decreased chondrocyte viability was noticed when concentration reached 200 μg/mL. Chondrocytes cultured with Ta-NH_2_ NPs did not manifest obvious morphology change (Figure 3I). Crystal violet staining, alcian blue staining, and immunofluorescent staining of COL-II and ACAN results indicated that Ta-NPs treatment did not affect the deposition of cartilaginous ECM (Figure 3I). However, western blot (Figures 3E, G) results showed that there were significant decreases in protein levels of COL-II, SOX-9, RUNX-2, and ADAMTS-5. Subsequent quantitative analysis further supported these results (Figures 3F, H). This observation suggests that small amount of ROS has a positive significance for the physiological regulation of cells under normal physiological conditions (Sies and Jones, 2020), Ta-NH_2_ NPs may break the ROS balance after co-culture with normal chondrocytes. As for the specific reasons, it remains to be further explored.

### Ta-NH_2_ NPs protects viability and hyaline-like phenotype in chondrocyte under oxidative stress *in vitro*

**FIGURE 4:**
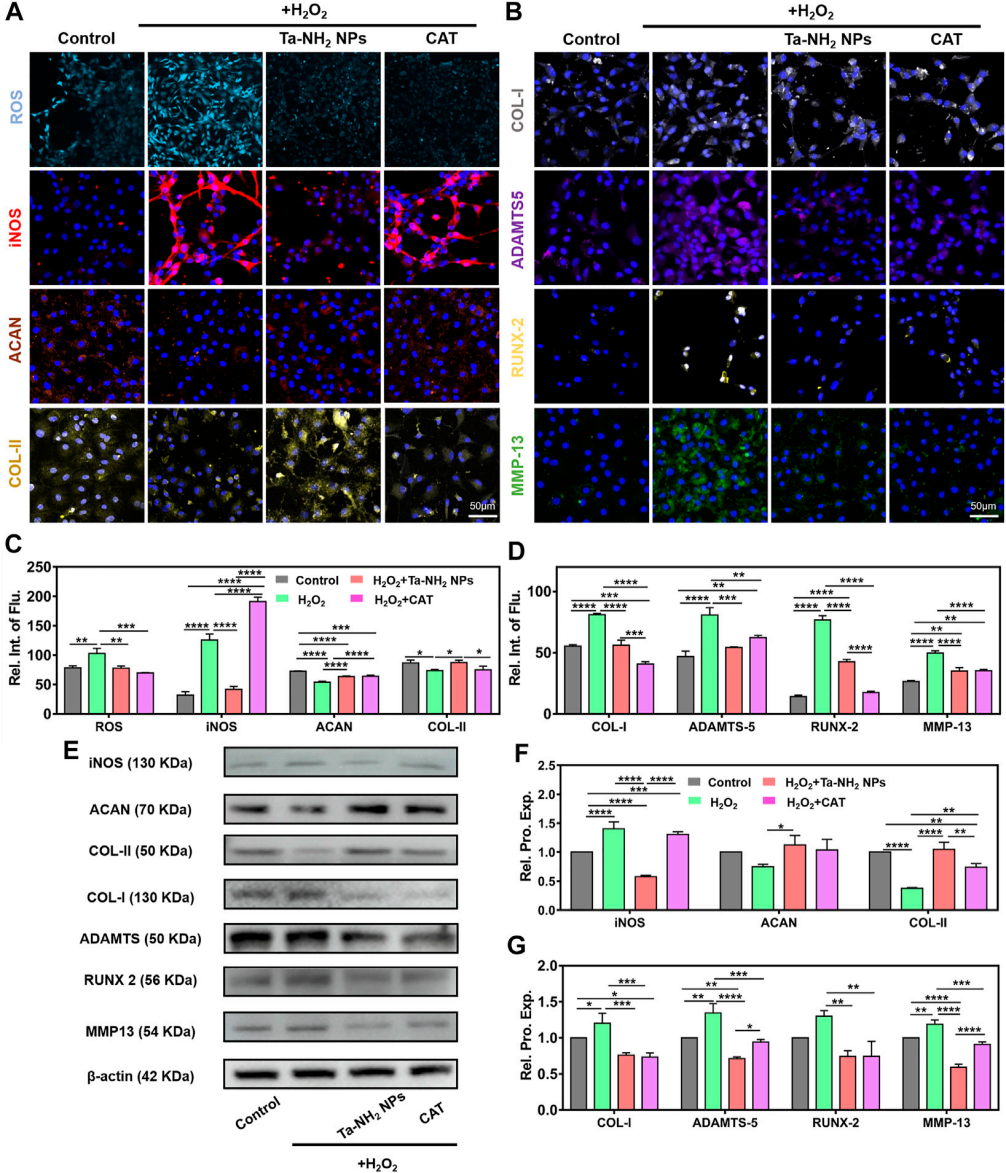
Ta-NH_2_ NPs and CAT reduced oxidative stress and maintained the chondrogenic phenotype of H_2_O_2_-stimulated chondrocytes. Fluorescence staining of **(A)** ROS, iNOS, ACAN and COL-II, **(B)** COL-I, ADAMTS-5, RUNX-2 and MMP-13 in H_2_O_2_ stimulated chondrocytes with or without Ta-NH_2_ NPs and CAT pretreatments. **(C,D)** Quantitative analyses of fluorescence intensity. **(E)** Western blot assay of protein expressions in above-described chondrocytes. **(F,G)** Quantitative analyses of protein expressions. Data were represented as means ± s.d, from three independent replicates. *p < 0.05, **p < 0.01, ***p < 0.001, ****p < 0.0001.

During the progression of OA, iNOS from OA-affected cartilage may contribute to the inflammation and pathogenesis of cartilage destruction (Yang et al., 2020). Chondrocytes showed over expression of iNOS mainly in the superficial zone in unhealthy OA cartilage (Amin et al., 1995). Expression of iNOS could reflect the degree of oxidative stress (Yang et al., 2020). To investigate the inhibitory effect of Ta-NH_2_ NPs on iNOS and ROS production in chondrocytes under oxidative stress, we pre-treated cells with Ta-NH_2_ NPs (100 μg/mL) or catalase (CAT, 100 μg/mL) for 1 h. Cells were then challenged with H_2_O_2_ for 24 h (400 μM). DCFH-DA staining indicated significant increase in intra-cellular ROS level in H_2_O_2_ treated group (Figure 4A), which was inhibited by either Ta-NH_2_ NPs or CAT. In agreement with previous studies, our data showed significant increase in iNOS expression post H_2_O_2_ challenge. While pre-treatment with Ta-NH_2_ NPs, but not CAT reversed the iNOS level (Figure 4B). In addition, live and dead staining data (Supplementary Figure S2B) indicated Ta-NH_2_ NPs or CAT pre-treatment successfully protected chondrocytes viability *via* inhibiting intra-cellular ROS production. These data suggested that although Ta-NH_2_ NPs and CAT showed similar protective effect under oxidative stress, different mechanism might be involved.

In the aspect of phenotypic alternation, our immunofluorescence staining and Western blot results (Figures 4A, B, E) suggested that H_2_O_2_ significantly decreased the hyaline-like phenotype in chondrocytes, while increased fibrotic (COL-I) and catabolism (ADAMTS-5, RUNX-2, and MMP-13) markers (Charlier et al., 2019; Lian et al., 2019). Further quantitative analysis suggested the similar results (Figures 4C, D, F, G). It is worth mentioning that fibrosis and hypertrophy are important pathological change in osteoarthritic cartilage (Yang et al., 2020; Yang et al., 2022). Previous studies have revealed that reduction of oxidative stress attenuated fibrosis and hypertrophy indexes effectively in tissue-organ level, including liver (Xu et al., 2022), kidney (Liu Z. et al., 2020), and heart (Dey et al., 2018). Similar to CAT, Ta-NH_2_ NPs inhibited the increment in protein levels of COL-I, RUNX-2, and MMP-13 (Figure 4E), and maintained the hyaline-like phenotype (ACAN and COL-II) in H_2_O_2_-treated chondrocytes. Compared with CAT group, it is worth noticing that Ta-NH_2_ NPs group showed more significant increase in COL-II, and decrease in ADAMTS-5 and MMP-13 protein levels. The observation indicated that Ta-NH_2_ NPs were more capable of restoring the balance between catabolism and anabolism. This effect of Ta-NH_2_ NPs might be attributed to the inhibitory ability of Ta-NH_2_ NPs in iNOS expression. Previous study suggested that NO promoted degradation of ECM by enhancing the activity of matrix metalloproteinase (MMPs) that subsequently led to joint destruction (Lepetsos and Papavassiliou, 2016). H_2_O_2_ leads to chondrocyte apoptosis by disrupting mitochondria, reducing mtDNA integrity and repair capacity. Small-sized Ta NPs could enter cell plasma, and possibly into mitochondria (Supplementary Figure S3A), reducing the negative effect of hydrogen peroxide on mitochondria through redox properties. In arthritic tissues, the NF-κB signaling pathway can be activated by pro-inflammatory cytokines, mechanical stress, and ECM degradation products. The NF-κB signaling pathway affects cartilage matrix remodeling (Arra et al., 2020), chondrocyte apoptosis, synovial inflammation, and has an indirect stimulating effect on downstream regulators of terminal chondrocyte differentiation. The interaction between redox signaling and NF-κB transcription factors appears to play a unique role in the pathogenesis of OA (Yang et al., 2020). Activation of NF-κB inhibits chondrocyte anabolism and triggers the expression of multiple matrix-degrading proteases, such as metalloproteases and ADAMTS, leading to erosion of articular cartilage (Deng et al., 2018). In addition, the expression of iNOS is also strongly related to NF-κB, which enhances the production of synovial iNOS, produces a large amount of NO, and mediates the expression of OA synovial inflammatory factors. Combined with our results, in addition to relying on its redox in mitochondria, Ta NPs might maintain the cell state by affecting the NF-κB pathway.

### *In vivo* biodistribution and biocompatibility assessment of Ta-NH_2_ NPs

**FIGURE 5:**
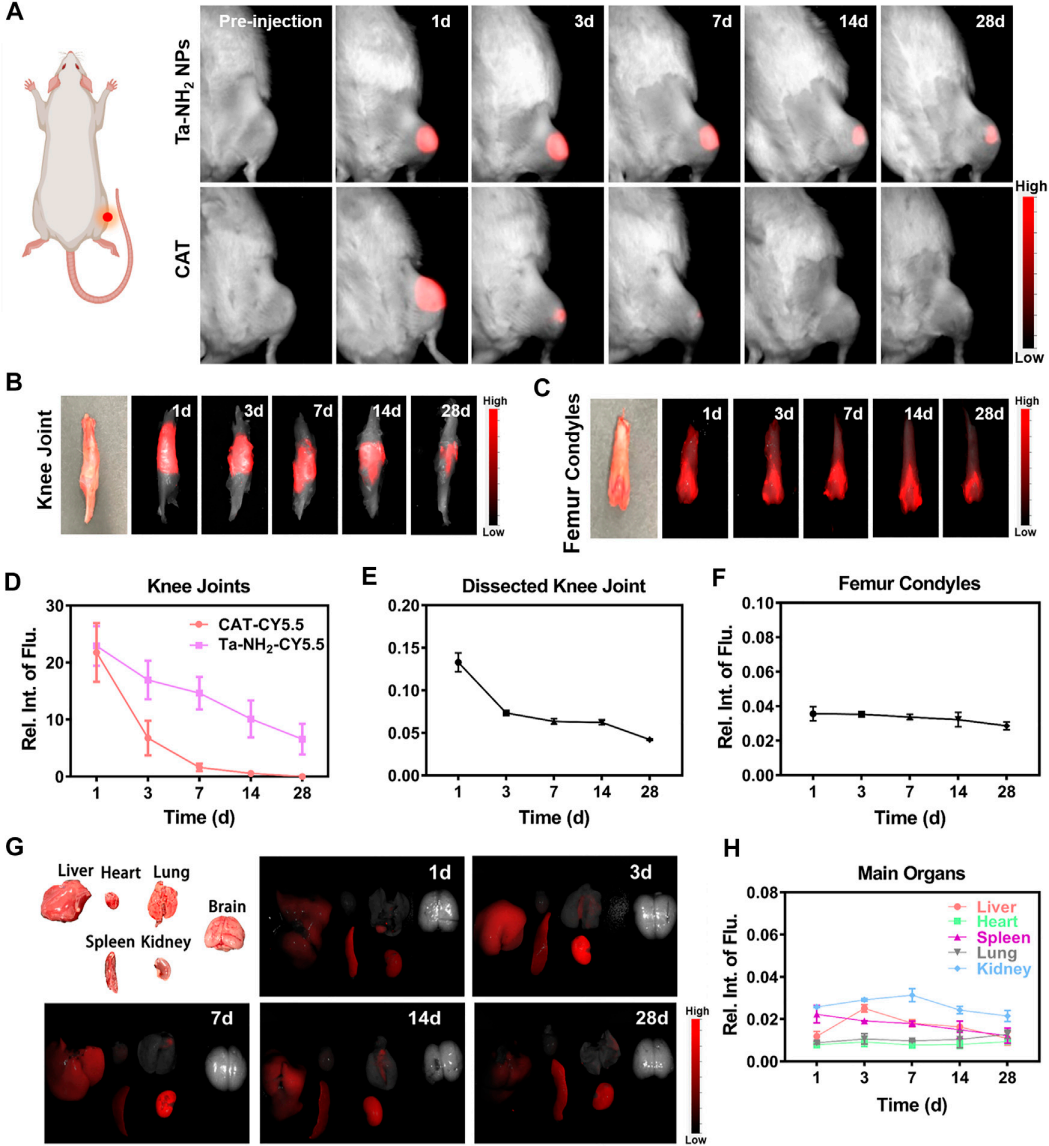
Metabolic kinetics of Ta-NH_2_ NPs in knee joints and biodistribution in main organs of SD rats. *In vivo* fluorescence imaging of cy5.5-labelled Ta-NH_2_ NPs and CAT in **(A)** intact, cy5.5-labelled Ta-NH_2_ NPs in **(B)** dissected rat knee joints, and **(C)** femoral condyles from 1 to 28 days post single intra-articular injection. Fluorescence quantifications of intact **(D)** and dissected **(E)** knee joints and femoral condyles **(F)** post intra-articular injection. **(G)** Biodistribution of Ta-NH_2_ NPs and corresponding **(H)** fluorescence quantification in major organs during 1–28 days post single intra-articular injection. Data were represented as means ± s.d., from three independent replicates.

Currently, drug delivery by intra-articular injection suffers from short retention in the joint cavity (Brown et al., 2019). Small molecules are rapidly cleared from the joint cavity within hours *via* synovial vasculature, and macromolecules within days *via* synovial lymphatics (Gerwin et al., 2006). In order to achieve stable pharmacokinetics, increased dosage or repetitive injection are often necessary (Gammer and Brobäck, 1984). In addition to biocompatibility, the ideal therapeutic antioxidant should also have high efficiency and long-term effect. In the present study, the positively charged NPs were designed to prolong the retention of Ta-NPs in articular cartilage. As shown in Figure 5A, after intra-articular injection of Cy5.5 labelled Ta-NH_2_ NPs (100 μg/mL) and CAT (100 μL), *in vivo* fluorescence imaging indicated that gradually decreased concentration of both antioxidants at day 1, 3, 7, 14, and 28. Whereas the Cy5.5 dye alone showed rapid decrease in fluorescence intensity 1 h post injection (Supplementary Figure S4A). Compared with CAT group (at day 3), significantly longer joint retention (at day 28) of Ta-NH_2_ NPs was noticed. In addition, further analysis in dissected knee joints confirmed that Cy5.5 labelled Ta-NH_2_ NPs, but not Cy5.5 labelled CAT (Supplementary Figure S4B) was still detectable 28 d post injection in femoral articular cartilage (Figures 5B, C). Agreed with previous study, these data confirmed the positively charged Ta NPs were able to stay in the joint cavity, particularly in the articular cartilage. From the imaging of the dissected femoral condyle, sustained fluorescence over 28 d observational period outlined the shape of trochlear cartilage, indicating that Ta-NPs after amino modification could be adsorbed in the surface of the articular cartilage.

**FIGURE 6:**
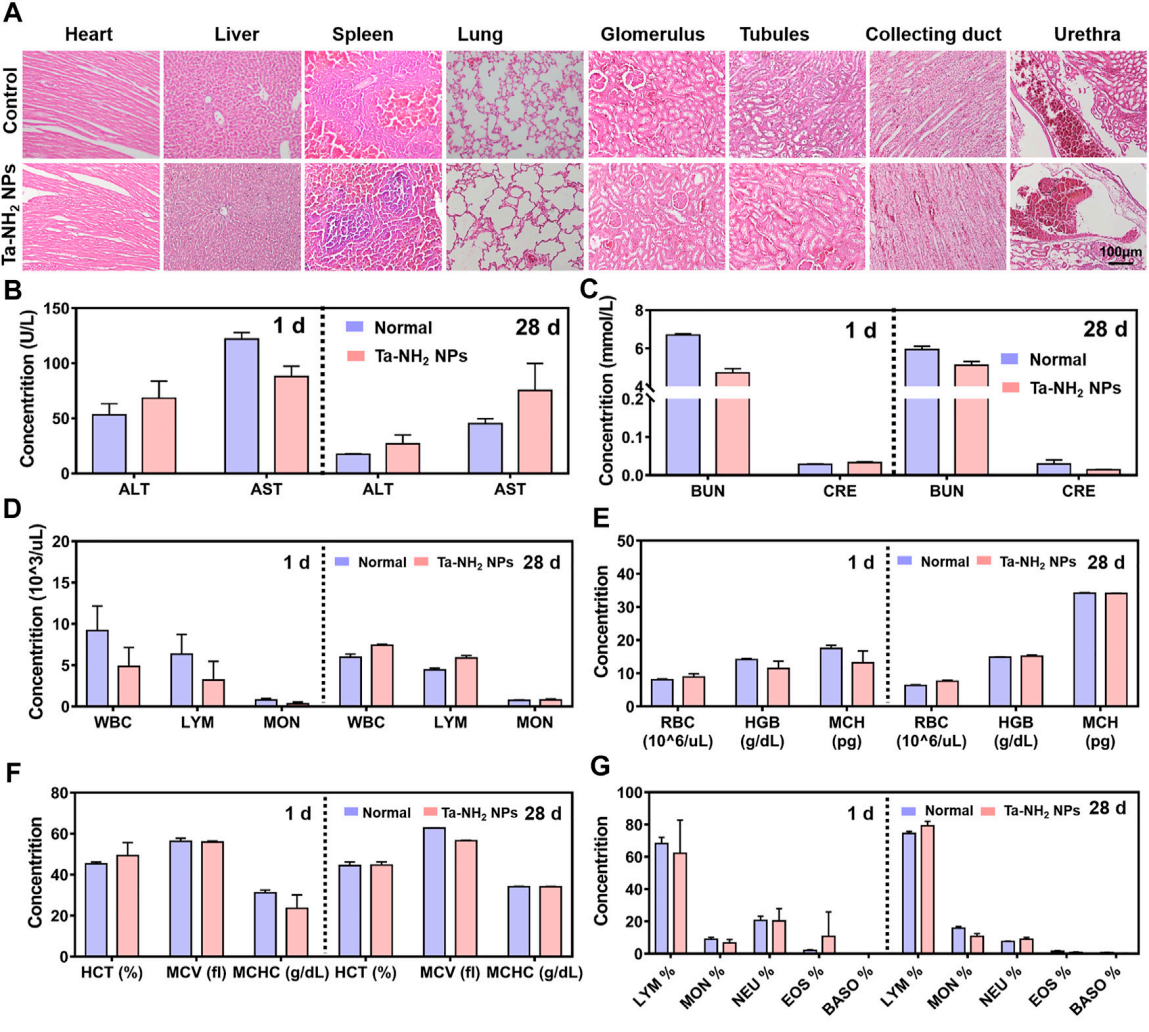
*In vivo* biocompatibility assessment of Ta-NH_2_ NPs. **(A)**. Evaluation of *in vivo* toxicity of Ta-NH_2_ NPs to major organs (heart, liver, spleen, and lung) at 1 d after knee joint injection. Serum levels of liver function indicators: aspartate aminotransaminase (AST) and alanine aminotransaminase (ALT) 1 d and 28 d after injection **(B)**. Serum levels of kidney function indicators: blood urea nitrogen (BUN) and creatinine (CRE) 1 d and 28 d after injection **(C)**. **(D–G)** Blood parameters in normal rat and rat knee joint injected with Ta-NH_2_ NPs 1 d and 28 d after injection. In **(B–E)**, data were represented as means ± s.d., from three independent replicates.

To further explore the biocompatibility and potential organ toxicity of Ta-NH_2_ NPs, we analyzed the biodistribution in main organs. Liver, heart, spleen, lung, kidney, and brain from rat post intra-articular injection of Cy5.5 labelled Ta-NH_2_ NPs were tested. No hemorrhage, atrophy, or necrosis was found in the analyzed organs. At the macroscopic level, Ta-NH_2_ NPs were mainly accumulated in the liver, spleen and kidney. Among them, the fluorescence intensity in spleen, liver and kidney reached the peak at day 1, 3, and 7 post injection and decayed by day 3, 7, and 14 respectively. This observation suggested that Ta-NH_2_ NPs in the organ might be gradually metabolized. To evaluate the organ toxicity of Ta-NH_2_ NPs, histological analysis was employed. H&E staining of main organs indicated no necrosis, congestion, or hemorrhage in the heart, liver, spleen, and lung at day 1, 3, 7, 14, and 28 (Supplementary Figure S4) after single dose intra-articular injection of Ta NPs (Figure 6A). Moreover, no distinguishable inflammatory, lesion or tissue damage was observed in the glomerulus, tubules, collecting ducts, and urethra, illustrating the excellent biocompatibility of Ta-NH_2_ NPs. To further explore functional changes of these organs, the hemogram (Figures 6D–G–G) and blood biochemistry analysis was performed. We selected AST and ALT, CRE and BUN to reflect the liver and renal functions, respectively. Compared with control group, our data showed no significant alternation in the size, number, and composition of blood cells, and in AST, ALT (Figure 6B), BUN, and CRE levels (Figure 6C).

### *In vivo* therapeutic effect of Ta-NH_2_ NPs in MIA induced OA model

**FIGURE 7:**
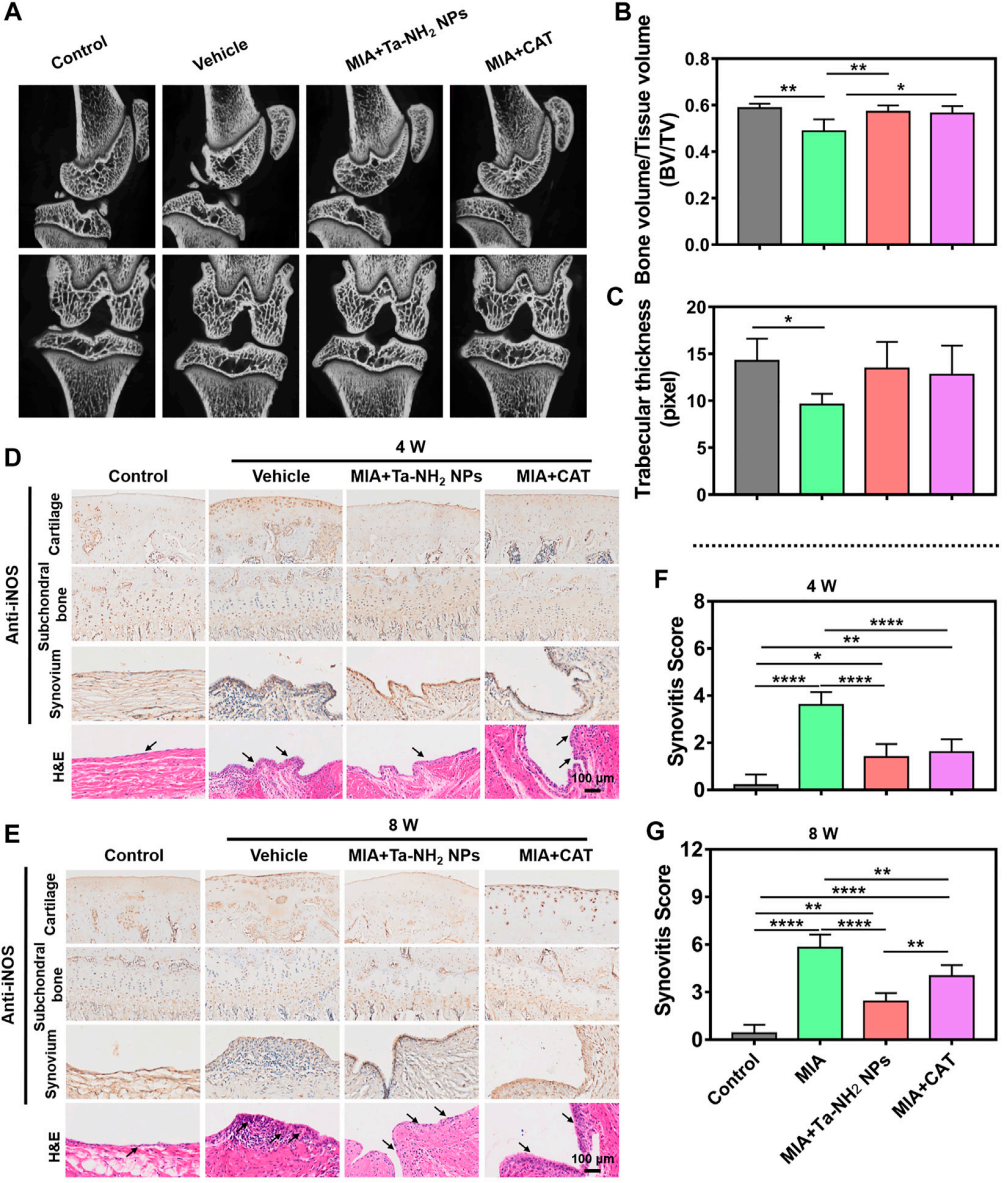
Ta-NH_2_ NPs restored MIA-induced bone structure and alleviated the inflammation in synovium in rat model. **(A)** Micro-CT images of the MIA pretreated knee joints after single articular injection of Ta-NH_2_ NPs or CAT. **(B,C)** Statistic analysis of Bone value/Tissue value and Trabecular thickness. **(D,E)** iNOS immunohistochemical and H&E staining of articular cartilage, subchondral bone, synovium and H&E staining of synovial tissue at 4 and 8 weeks post treatments. **(F,G)** Histopathological scoring of synovium at 4 and 8 weeks post treatments. The arrow points to the synovium and hyperplasia blood vessels. Data were represented as means ± s.d., from three or five independent replicates (One-way ANOVA). *p < 0.05, **p < 0.01, ***p < 0.001, ****p < 0.0001.

As shown in Supplementary Figure S6, the rat OA model was induced by MIA injection, followed by the corresponding antioxidant injection. At 8 weeks post injection, compared with that in the sham operation group, the micro-CT scanning indicated obvious bone defects in the patella and femoral condyle, and alternation in subchondral bone structure in vehicle group (Figure 7A, white arrows). The subchondral bone structure was significantly improved by Ta-NH_2_ NPs or CAT injection (*p* < 0.05, Figures 7B, C).

In order to verify the long-term anti-oxidative stress effect of Ta-NH_2_ NPs post single intra-articular injection, iNOS expression was detected in articular cartilage, subchondral bone and synovium during the entire process (Figures 7D, E). The expression of iNOS in articular cartilage was significantly increased, and the expression reached the peak at the 8th week after MIA injection (Figure 7E). Antioxidant treatment decreased the expression of iNOS, particularly in Ta-NH_2_ NPs treated group. In addition, Ta-NH_2_ NPs, but not CAT showed prolonged inhibition of iNOS expression at the 8th week after MIA injection (Figure 7E). Cartilage is the main source of NO in OA (Melchiorri et al., 1998), and iNOS expression is more enhanced in chondrocytes compared with synovial cells from patients with OA (Melchiorri et al., 1998). In addition, expression of iNOS is mainly concentrated in the superficial osteoarthritic cartilage region (Amin et al., 1995), whereas chondrocytes isolated from patients without OA do not express iNOS. Our data were in line with these observations, in which we showed iNOS expression was mainly confined to articular cartilage, especially in the superficial zone. The cartilage-targeting ability of Ta-NH_2_ NPs can penetrate through cartilage (Supplementary Figures S3B, C) and might act through direct oxidation resistance in the ECM, or reduce intracellular iNOS synthesis *via* phagocytosis. The iNOS expression in synovium tissue was similar to cartilage, whereas no significant difference in iNOS expression in subchondral bone was noticed (Figures 7D, E).

The synovium is a thin connective tissue that attaches to the joints, its inflammation is mediated by activation of mitochondrial dysfunction (Sanchez-Lopez et al., 2022), cytokines, and metabolites in synovial cells. The inter-communication between chondrocytes and synovial cells is thought to be significant to joint homeostasis (Kurowska-Stolarska and Alivernini, 2022). Once synovitis is activated, cartilage undergoes subsequent adverse changes. In addition to the immunohistochemical staining of iNOS, we further observed synovial inflammation and angiogenesis by H&E staining (Figures 7D, E). It was not difficult to notice the MIA-induced synovial inflammation. In vehicle group, obvious hyperplasia and inflammatory cell infiltration were seen in synovium at 4 weeks post intra-articular injection. In addition, vessel hyperplasia was also noticed at 8 weeks. The inflammation of the synovium was alleviated after both anti-oxidative treatments. Notably, synovitis score indicated synovial inflammation in the Ta-NH_2_ NPs group was significantly lower than that in the CAT group at 8 weeks post injection (Figures 7F, G).

**FIGURE 8:**
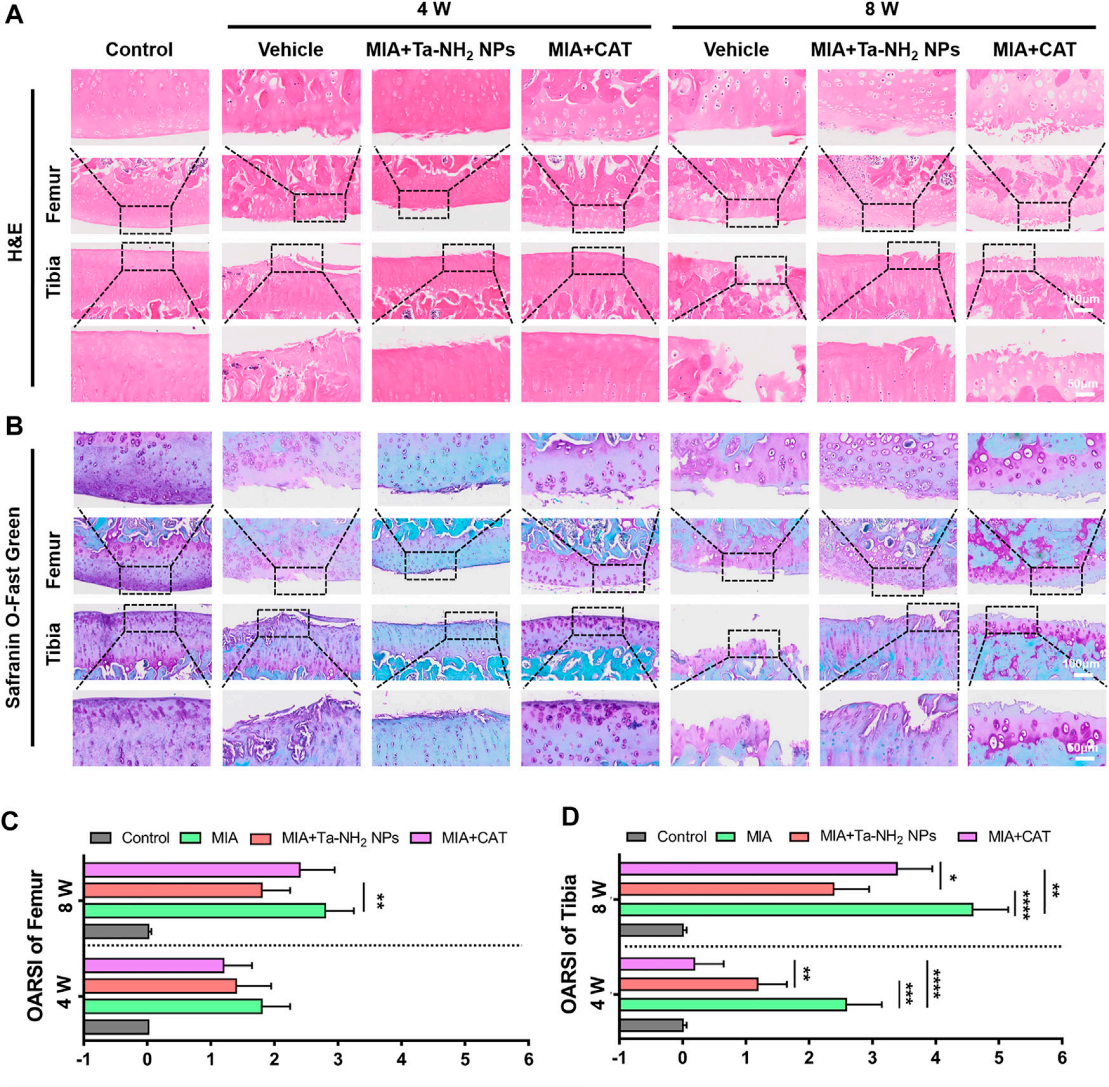
Therapeutic efficiency of Ta-NH_2_ NPs on MIA-induced OA. **(A)** H&E staining and **(B)** Safranin-O-Fast Green Staining of articular joints from each group. The box marked local magnification. OARSI score of **(C)** femur and **(D)** tibia at 4 and 8 weeks post treatments. Data were represented as means ± s.d., from five independent replicates (One-way ANOVA), *p < 0.05, **p < 0.01, ***p < 0.001, ****p < 0.0001.

The further evaluation of the therapeutic effect of Ta-NH_2_ NPs in MIA-induced OA model was based on H&E and safranin-o-fast green staining. As shown in Figures 8A, B, the vehicle group presented typical OA features such as surface irregularity, decreased expression of glycosaminoglycans, and cartilage defects. These cartilage damages were alleviated after antioxidant treatment. The Ta-NH_2_ NPs treatment presented sustained remission at week 8, while CAT treatment did not exert its antioxidant function at this time point, which was consistent with previous results. Compared with vehicle group, both antioxidants attenuated the OARSI score in femur, and significantly decreased the OARSI score in tibia (Figures 8C, D). In addition, Ta-NH_2_ NPs exhibited excellent long-term therapeutic effect as evidenced by more homogeneous glycosaminoglyc and cell arrangement (*p* < .05 when compared with CAT group).

## Conclusion

To overcome the disadvantages of current antioxidative strategy for OA, the present study designed and prepared positively charged Ta NPs with sustained intra-articular catalase activity. The *in vitro* results showed that our designed Ta-NH_2_ NPs had good biocompatibility and stability, and protected viability and hyaline-like phenotype in chondrocyte under oxidative stress. Our hypothesis was agreed with *in vivo* biodistribution data that Ta-NH_2_ NPs showed sustained retention in the joint cavity, particularly in articular cartilage. Finally, Ta-NH_2_ NPs exhibited long-term anti-oxidant stress and therapeutic effects in MIA-induced OA model. This study proposes a promising cartilage-protective approach to reduce oxidative stress in OA cartilage. Ta-NH_2_ NPs were designed to retain in cartilaginous ECM without inducing cytotoxicity or adverse reactions *in vitro* and *in vivo*. Given their joint retention time and ROS clearance capacity, these NPs might be used to treat or prevent OA. Our study provides a promising strategy for antioxidant therapy in OA, which is expected to be translated into clinical application in the future.

## Data Availability

The original contributions presented in the study are included in the article/Supplementary Material, further inquiries can be directed to the corresponding authors.
