# Supplementary material for: Sustained intra-articular reactive oxygen species scavenging and alleviation of osteoarthritis by biocompatible amino-modified tantalum nanoparticles
Source: Front Bioeng Biotechnol. 2023 Jan 13;11:1118850. doi: 10.3389/fbioe.2023.1118850 (PMC9880278; doi:10.3389/fbioe.2023.1118850)
Supplement: Supplementary file 1 [file DataSheet1.PDF]

Supplementary Material

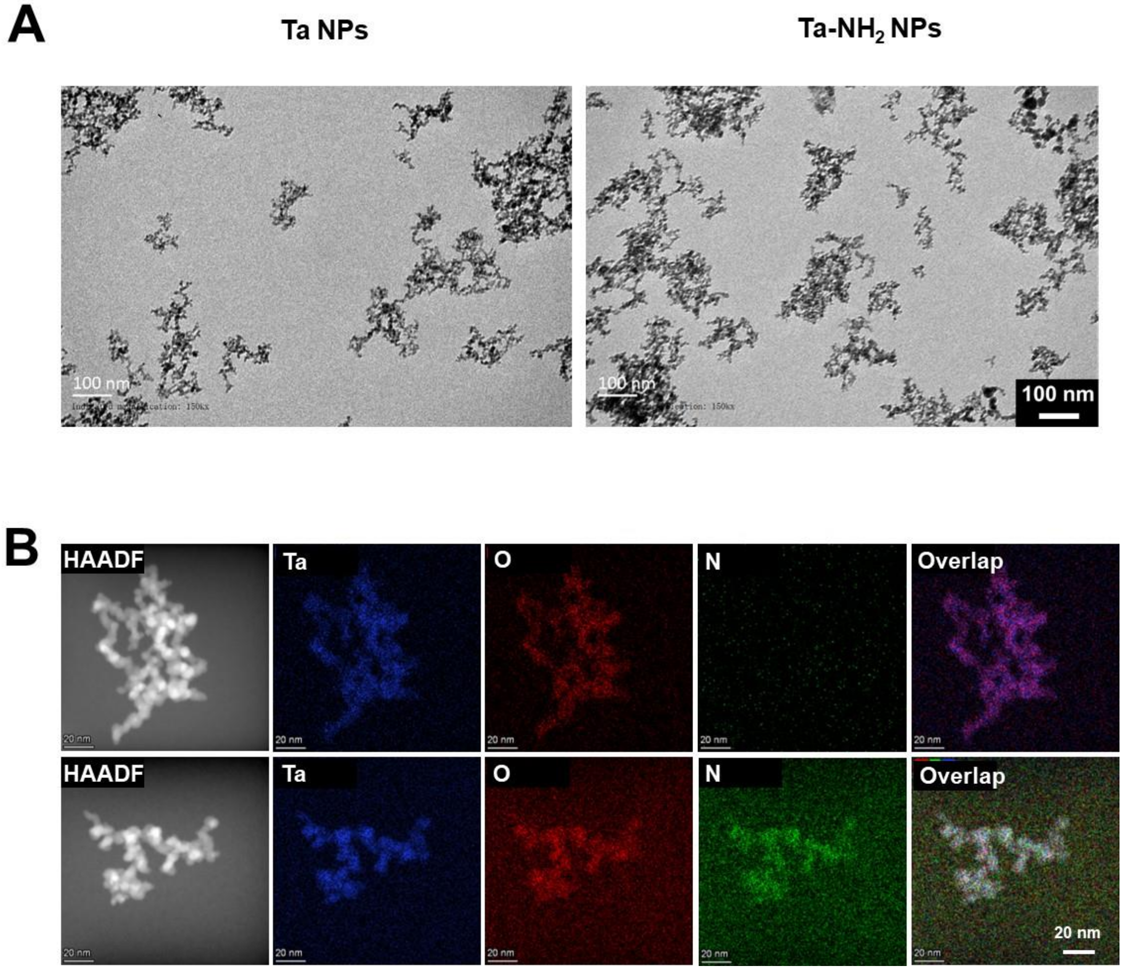

Figure S1. **(A)** TEM images of Ta NPs and Ta-NH2 NPs. **(B)** Dark-field TEM image of Ta NPs and Ta-NH2 NPs and corresponding TEM elemental mappings.

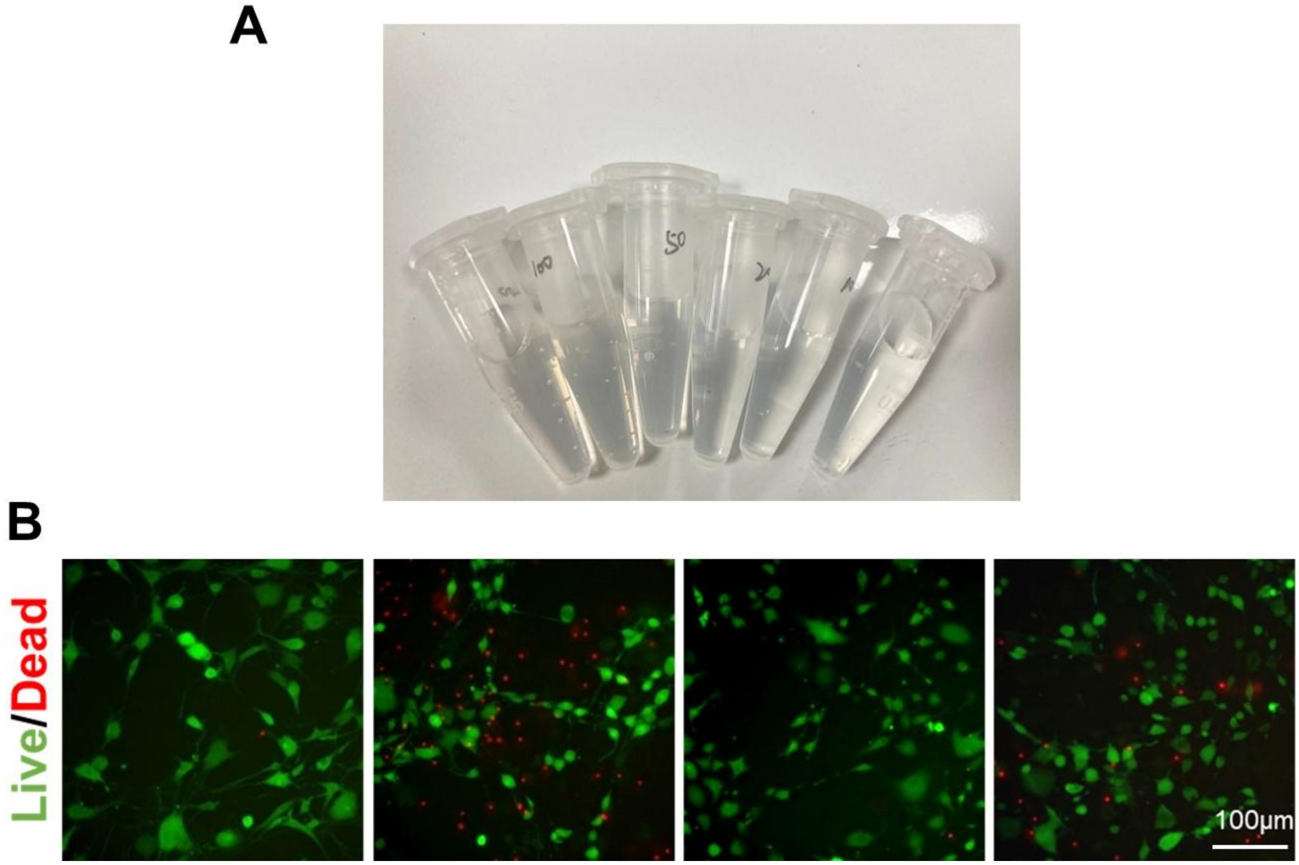

Figure S2. (A) Bubbles were generated after adding Ta-NH_2 NPs into H_2O_2 solution. **(B)** Live/dead staining of chondrocytes in different culture environment.

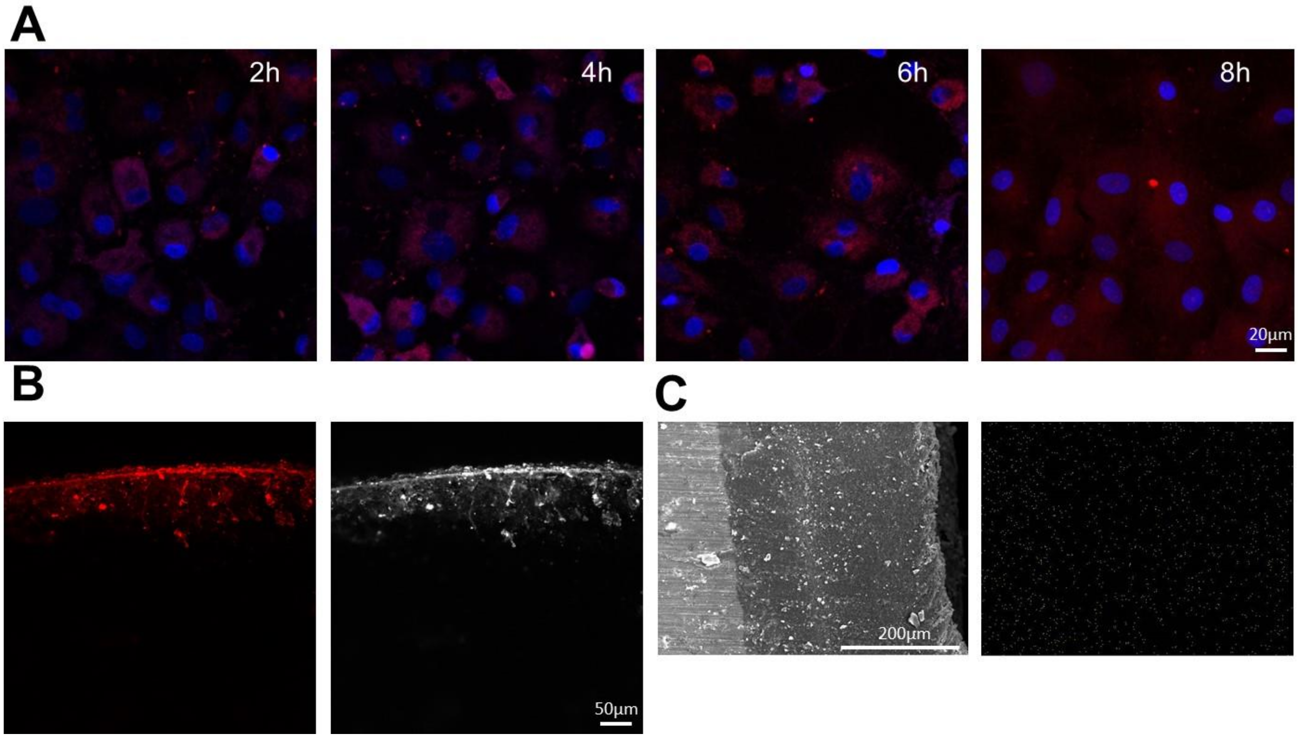

Figure S3. **(A)** Cy5.5-labeled Ta-NH_2 NPs were phagocytosed by chondrocytes. **(B)** The affinity of Ta-NH_2 NPs to articular cartilage was observed by confocal microscope and **(C)** the distribution of tantalum in cartilage was observed by scanning electron microscope

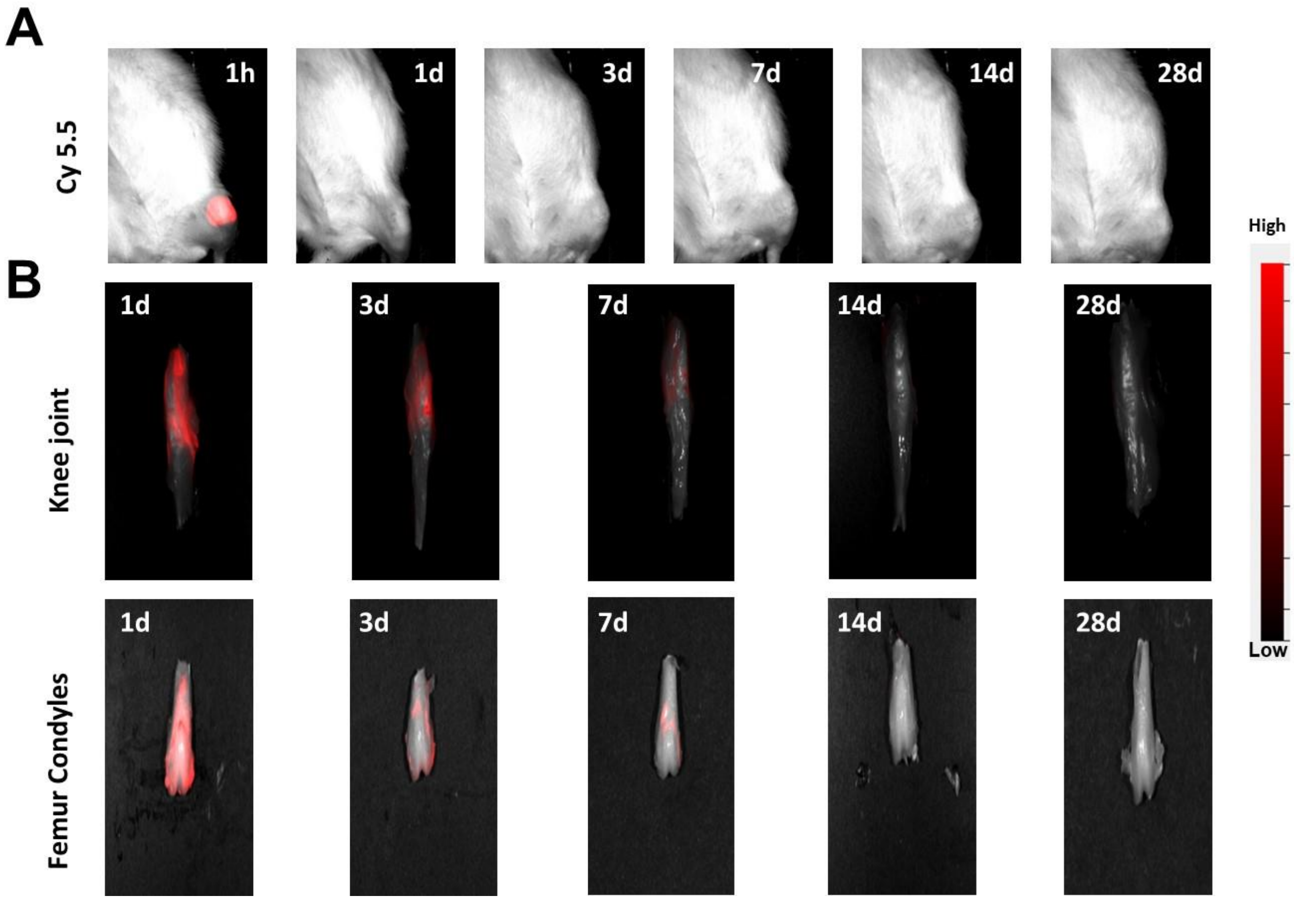

Figure S4. **(A)** *In vivo* detection in fluorescence intensity of cy5.5 in rat knee joints for 28 d. (**B)** Knee and femoral condyle fluorescence reimaging of cy5.5 labeled CAT at 1-28 d.

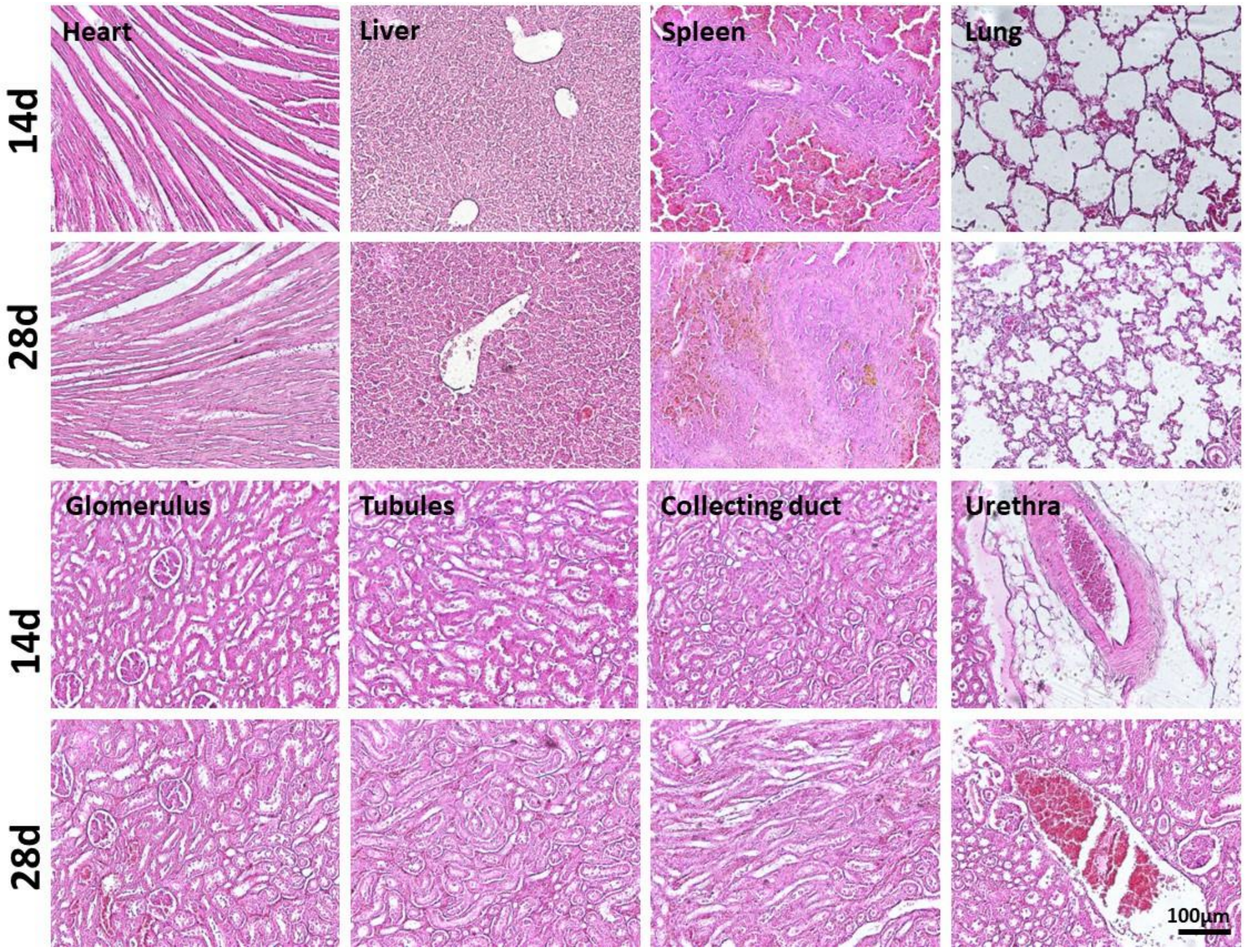

Figure S5. Evaluation of *in vivo* toxicity of Ta-NH_2 NPs to major organs (heart, liver, spleen, and lung) at 14d and 28d post single intra-articular injection.

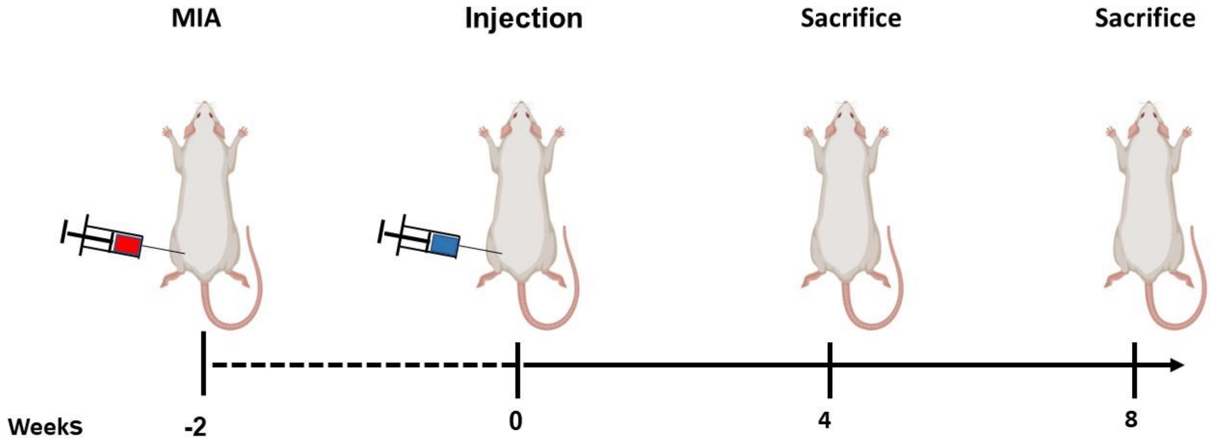

Figure S6. Schematic illustration of MIA-induced OA in SD rat
